# Optical Ultrastructure of Large Mammalian Hearts Recovers Discordant Alternans by *In Silico* Data Assimilation

**DOI:** 10.3389/fnetp.2022.866101

**Published:** 2022-04-13

**Authors:** Alessandro Loppini, Julia Erhardt, Flavio H. Fenton, Simonetta Filippi, Marcel Hörning, Alessio Gizzi

**Affiliations:** ^1^ Nonlinear Physics and Mathematical Modeling Laboratory, University Campus Bio-Medico of Rome, Rome, Italy; ^2^ Biobased Materials Laboratory, Institute of Biomaterials and Biomolecular Systems, Faculty of Energy, Process and Biotechnology, University of Stuttgart, Stuttgart, Germany; ^3^ School of Physics, Georgia Institute of Technology, Atlanta, GA, United States

**Keywords:** cardiac alternans, cardiac arrhythmias, optical mapping, frequency analysis, mathematical modeling, data assimilation

## Abstract

Understanding and predicting the mechanisms promoting the onset and sustainability of cardiac arrhythmias represent a primary concern in the scientific and medical communities still today. Despite the long-lasting effort in clinical and physico-mathematical research, a critical aspect to be fully characterized and unveiled is represented by spatiotemporal alternans patterns of cardiac excitation. The identification of discordant alternans and higher-order alternating rhythms by advanced data analyses as well as their prediction by reliable mathematical models represents a major avenue of research for a broad and multidisciplinary scientific community. Current limitations concern two primary aspects: 1) robust and general-purpose feature extraction techniques and 2) *in silico* data assimilation within reliable and predictive mathematical models. Here, we address both aspects. At first, we extend our previous works on Fourier transformation imaging (FFI), applying the technique to whole-ventricle fluorescence optical mapping. Overall, we identify complex spatial patterns of voltage alternans and characterize higher-order rhythms by a frequency-series analysis. Then, we integrate the optical ultrastructure obtained by FFI analysis within a fine-tuned electrophysiological mathematical model of the cardiac action potential. We build up a novel data assimilation procedure demonstrating its reliability in reproducing complex alternans patterns in two-dimensional computational domains. Finally, we prove that the FFI approach applied to both experimental and simulated signals recovers the same information, thus closing the loop between the experiment, data analysis, and numerical simulations.

## 1 Introduction

In nature, a broad variety of pattern formations can be found on very different length scales and functions, such as collective behavior of fish swarms (Jakobsen and Johnsen, 1988), the animal skin patterning (Murray, 2003; Miyazawa et al., 2010), the cell dynamics during embryogenesis (Ju et al., 2017), the formation of neuronal networks in brains (van den Heuvel and Hulshoff Pol, 2010), and the electromechanical function of the cardiovascular system (Christoph et al., 2018). The latter is crucial to maintain life as we know but is susceptible to malfunction due to its complex morphology such as the vascular system (Luther et al., 2011), cellular orientation (Papadacci et al., 2017), and mechano-electrophysiological wave patterning (Hörning et al., 2012). Slight variations in the organization of those patterns can have fatal consequences, and thus, cardiovascular diseases are the primary cause of death in industrial countries.

One of the complex and not fully understood heart behaviors, possibly inducing cardiac disease, is alternans. It describes a phase-dependent alternation on either a single-cell or tissue level and can be described as a beat-to-beat alternation of short and long heartbeats (membrane potential, intracellular calcium) or myocyte contractions. Alternans is known to be involved in a series of cardiovascular conditions as either cause or symptom. These include, among others, ventricular fibrillation, arrhythmias, and sudden cardiac death (Adam et al., 1984; Konta et al., 1990; Pastore et al., 1999), especially in patients after myocardial infarction (Ikeda et al., 2000). Other triggers for alternans are ischemia of the myocardium, ectopic heartbeats, and coronary occlusion (Green, 1935; Dilly and Lab, 1988; Taggart et al., 1996; Ren et al., 2011). In early studies, alternans was observed in terms of myocardial contractility, aortic pressure, and stroke volume (Mitchell et al., 1963). In medical applications, this phenomenon has therefore been widely employed as a predictive tool for determining risks for fibrillation, venous thromboembolism, arrhythmia, and sudden cardiac death (Dilly and Lab, 1988; Kim et al., 2009). Besides, it is used to assess the necessity and urgency of certain surgical operations, such as implantation of cardioverter defibrillators (Merchant et al., 2012).

Several mechanisms have been revealed during three decades of intensive research that can induce alternans. Early studies stated the critical role of calcium cycling and electrical restitution of the action potential in the generation of alternans (Badeer et al., 1967; Dilly and Lab, 1988; Konta et al., 1990). Repolarization gradients at the tissue level have further been shown to lead to complex macroscopic voltage alternans patterns (Pastore et al., 1999). Later, it was shown that fluctuations in the cyclic release of Ca^2+^ from the sarcoplasmic reticulum could lead to Ca^2+^ alternans tightly coupled with voltage repolarization alternans (Lab and Lee, 1990; Qu et al., 1999; Walker et al., 2003; Diaz et al., 2004). Similar to that, a fine-scaled initiation of alternans was linked to the subsequent formation of larger alternating regions (Jia et al., 2010). Additionally, alternans can be promoted by low temperature or application of drugs (Xie and Weiss, 2009; Gizzi et al., 2017; Loppini et al., 2021).

While alternans can be observed at single cells for the action potential duration (APD) and the calcium transient amplitude (CTA), in tissue, those oscillations can synchronize and lead to spatial concordant alternans (CA) or discordant alternans (DA) (Uzelac et al., 2017). CA is observed when the entire tissue alternates in phase, while DA is classified with at least two out-of-phase oscillating regions (Xie and Weiss, 2009; Gizzi et al., 2013; Gizzi et al., 2017) spatially separated by nodal lines, i.e., non-alternating domains (Hörning et al., 2017). The conduction velocity (CV) plays a crucial role in developing alternans. Usually, concordant or discordant APD and CTA depend on CV restitution (Karagueuzian et al., 2013). A slowing of the CV, caused by the incomplete recovery of the fast sodium current, concurs to promote large gradients of repolarization, thus sustaining DA patterns. Furthermore, alternans can be studied in terms of electromechanically out-of-phase regions. In this case, larger CTAs are triggered by shorter APDs and vice versa (Sato et al., 2006).

Based on the experimental finding, numerous computational models have been developed that can show the onset and transition of alternans in both single cells and tissue (Karma, 1993, 1994; Qu et al., 1999; Watanabe et al., 2001; Cherry and Fenton, 2004; Tao et al., 2008; Shiferaw et al., 2003; Huertas et al., 2010). However, the striking limitation of the current modeling approaches consists in the capability of reproducing complex DA patterns in anatomically realistic computational domains. In practice, the appearance of CA and DA in numerical simulations requires, up to now, an *ad hoc* tuning of physiological parameters, usually deviating from the optimal set obtained from experimental CV and restitution curves (Cairns et al., 2017). Innovative multiscale and multiphysics formulations of cell–cell couplings aim at filling this gap. Non-linear, stress-assisted, and fractional diffusion (Lin and Keener, 2010; Hurtado et al., 2016; Cherubini et al., 2017; Cusimano et al., 2020; Cusimano et al., 2021), ephaptic and gap junction–mediated couplings (Lenarda et al., 2018; Weinberg, 2017), cellular automata, and coarse-grained homogenized gap junction approaches (Treml et al., 2021; Irakoze and Jacquemet, 2021) represent the state-of-the-art in this direction. Furthermore, within the specific context of cardiac electrophysiology, recent studies are proposing novel methods of data estimation, data assimilation, and uncertainty quantification (Barone et al., 2020a; Barone et al., 2020b; Pathmanathan et al., 2020; Marcotte et al., 2021) to reproduce complex cardiac dynamics with a reduced computational cost.

On such a ground, we propose an innovative data assimilation technique using the optical ultrastructure obtained from a frequency analysis of voltage fluorescence activations on intact canine ventricles demonstrating its potential role in recovering complex alternans patterns *in silico*. The results presented in this study fundamentally advance the understanding of alternans. Furthermore, the proposed observation strategy may enable possible applications to personalized medicine, such as quantifying alternans and higher-other rhythms without heavy computational resources or massive experimental campaigns. As the ultrastructure of the heart is unique for every subject, it may be used as the base for studying possible diseased states and treatments. Thus, this study lays the promising foundation for such approaches in the near future.

This manuscript is organized as follows. Section 2 introduces the Fourier-based method and the experimental data assimilation technique in electrophysiological mathematical models. Section 3 demonstrates the ability of our frequency technique to recover alternans in cardiac tissue at high-frequency pacing rates, and we identify the optical ultrastructure to assimilate *in silico*. Besides, the optimal combination of data assimilation heterogeneities matches complex experimental alternans patterns at best. Section 4 closes the work with a discussion of limitations and perspectives of the current approach.

## 2 Materials and Methods

### 2.1 Experimental Setup

Right ventricle wedges from canine were prepared according to the experimental protocols approved by the Institutional Animal Care and Use Committee of the Center for Animal Resources and Education at Cornell University. Fluorescence optical mapping recordings of the membrane potential were recorded at a spatial resolution of 600 × 600 μm^2^ per pixel for a grid size of 7.7 × 7.7 cm^2^ and a temporal resolution of 2 ms at physiological conditions. For details of the experimental setup information, we refer to the previous studies (Fenton et al., 2009; Luther et al., 2011; Gizzi et al., 2013; Gizzi et al., 2017).

### 2.2 Data Analysis

#### 2.2.1 Fourier Transformation Imaging

Fourier transformation imaging was applied to the fluorescence optical mapping recordings, as introduced before (Hörning et al., 2017). The optical recordings were pixel-wise decomposed and transformed to the mathematically complex Fourier space, *F*_*x*,*y*_(*f*), as a function of the frequency *f*, i.e.,

$$I_{x,y}\left(t\right)\to F_{x,y}\left(f\right)\text{,}$$ (1)

where *I*_*x*,*y*_(*t*) is the intensity at the spatial position (*x*, *y*) and *t* is the time. From that, the amplitude |*F*_*x*,*y*_(*f*)| and the phase arg(*F*_*x*,*y*_(*f*)) are calculated and spatially recomposed to two respective Fourier frequency-series.

#### 2.2.2 APD Alternans Maps

Alternans maps were pixel-wise calculated on pre-analyzed signals. Pre-analysis involves detrending, nearest-neighbor averaging in time with a rectangular window (7 frames width), and space filtering with Gaussian kernel (4 pixels radius). The APD is the extracted by threshold crossing at 20% $max\left(I_{x,y}\right)$ . The temporal difference of subsequent action potentials ΔAPD is then quantified as

$$\Delta {\text{APD}}_{n}={\text{APD}}_{n+1}-{\text{APD}}_{n}\text{,}$$ (2)

where *n* denotes the beat number. ΔAPD maps were recomposed, and a functional color scheme was applied that indicates non-alternating tissue and nodal lines when ΔAPD = 0 ± 2 ms, which is defined by the temporal resolution of the recordings. A larger or smaller ΔAPD shows phase-dependent alternans, as introduced before (Gizzi et al., 2013).

### 2.3 Mathematical Model

We based our numerical simulations on the four-variable minimal model for ventricular action potentials (Bueno-Orovio et al., 2008), solved on two-dimensional anisotropic heterogeneous spatial domains according to the fine-tuning performed by Fenton et al. (2013). The model includes a phenomenological description of main transmembrane ion currents and is properly generalized with a heterogeneous diffusion contribution to account for spatial effects. The model’s equations are

$$\partial_{t}u=\nabla \cdot \left(D_{ij}\nabla u\right)-\left(J_{fi}+J_{so}+J_{si}\right),$$ (3a)

$$d_{t}v=\left[1-\Theta \left(u-\theta_{v}\right)\right]\left(v_{\infty }-v\right)/\tau_{v}^{-}-\Theta \left(u-\theta_{v}\right)v/\tau_{v}^{+},$$ (3b)

$$d_{t}w=\left[1-\Theta \left(u-\theta_{w}\right)\right]\left(w_{\infty }-w\right)/\tau_{w}^{-}-\Theta \left(u-\theta_{w}\right)w/\tau_{w}^{+},$$ (3c)

$$d_{t}s=\left[\left(1+\tanh\left(k_{s}\left(u-u_{s}\right)\right)\right)/2-s\right]/\tau_{s},$$ (3d)

where *u* is the dimensionless cell membrane potential, *v*, *w*, and *s* are gating variables regulating ion current activation, and Θ(*x*) denotes the Heaviside step function. *J*_*fi*_, *J*_*so*_, *J*_*si*_ represent fast-inward, slow-outward, and slow-inward transmembrane currents:

$$J_{fi}=-\Theta \left(u-\theta_{v}\right)\left(u-\theta_{v}\right)\left(u_{u}-u\right)v/\tau_{fi},$$ (4a)

$$J_{so}=\left[1-\Theta \left(u-\theta_{w}\right)\right]\left(u-u_{o}\right)/\tau_{o}+\Theta \left(u-\theta_{w}\right)/\tau_{so},$$ (4b)

$$J_{si}=-\Theta \left(u-\theta_{w}\right)ws/\tau_{si}.$$ (4c)

Time constants and asymptotic values for gating variables depend on the membrane voltage:

$$\tau_{v}^{-}\left(u\right)=\left[1-\Theta \left(u-\theta_{v}^{-}\right)\right]\tau_{v1}^{-}+\Theta \left(u-\theta_{v}^{-}\right)\tau_{v2}^{-},$$ (5a)

$$\tau_{w}^{+}\left(u\right)=\tau_{w1}^{+}+\left(\tau_{w2}^{+}-\tau_{w1}^{+}\right)\left[\tanh\left[k_{w}^{+}\left(u-u_{w}^{+}\right)\right]+1\right]/2,$$ (5b)

$$\tau_{w}^{-}\left(u\right)=\tau_{w1}^{-}+\left(\tau_{w2}^{-}-\tau_{w1}^{-}\right)\left[\tanh\left[k_{w}^{-}\left(u-u_{w}^{-}\right)\right]+1\right]/2,$$ (5c)

$$\tau_{so}\left(u\right)=\tau_{so1}+\left(\tau_{so2}-\tau_{so1}\right)\left[\tanh\left[k_{so}\left(u-u_{so}\right)\right]+1\right]/2,$$ (5d)

$$\tau_{s}\left(u\right)=\left[1-\Theta \left(u-\theta_{w}\right)\right]\tau_{s1}+\Theta \left(u-\theta_{w}\right)\tau_{s2},$$ (5e)

$$\tau_{o}\left(u\right)=\left[1-\Theta \left(u-\theta_{o}\right)\right]\tau_{o1}+\Theta \left(u-\theta_{o}\right)\tau_{o2},$$ (5f)

$$v_{\infty }=1-\Theta \left(u-\theta_{v}^{-}\right),$$ (5g)

$$w_{\infty }=\left[1-\Theta \left(u-\theta_{o}\right)\right]\left(1-u/\tau_{w_{\infty }}\right)+\Theta \left(u-\theta_{o}\right)w_{\infty }^{*}.$$ (5h)

*D*_*ij*_ is the two-dimensional diffusion tensor, defined as

$$D_{ij}\equiv \frac{\sigma_{ij}}{S_{o}\;C_{m}}=\left(\begin{array}{cc} D_{11} & D_{12}\\ D_{21} & D_{22} \end{array}\right),$$ (6)

where *σ*_*ij*_ is the conductivity tensor, *S*_*o*_ represents the cell surface-to-volume ratio, and *C*_*m*_ is the membrane capacitance. The tensor elements are defined in the two-dimensional Cartesian domain as

$$D_{11}=D_{∥}\cos^{2}\left(\alpha \left(x,y\right)\right)+D_{⊥}\sin^{2}\left(\alpha \left(x,y\right)\right),$$ (7a)

$$D_{22}=D_{∥}\sin^{2}\left(\alpha \left(x,y\right)\right)+D_{⊥}\cos^{2}\left(\alpha \left(x,y\right)\right),$$ (7b)

$$D_{12}=D_{21}=\left(D_{∥}-D_{⊥}\right)\cos\left(\alpha \left(x,y\right)\right)\sin\left(\alpha \left(x,y\right)\right).$$ (7c)

Here, the function *α*(*x*, *y*) represents the local fiber orientation and *D*_∥_ and *D*_⊥_ denote diffusivities along the directions parallel and perpendicular to the fibers. We used the same anisotropy settings as in previous computational studies on cardiac activation maps (Loppini et al., 2019). Model parameters are reported in Table 1. The parameter set is consistent with the one originally fine-tuned by Fenton et al. (2013), related to endocardial tissue at 37° Celsius. Specifically, parameter values are set to reproduce AP features, conduction velocity, and restitution curves as observed in canine cardiac tissues, the same considered in this study. Furthermore, we assume the anisotropy ratio as 1:3 in line with previous modeling analyses (Loppini et al., 2019). For a more detailed description of model parameters and for a comparison between the four-variable model results and experiments, we refer the reader to the two abovementioned studies.

**TABLE 1:** Model parameters for the ventricular action potential. The membrane voltage *u* and related parameters are dimensionless. The scaling *u*_*m*_ = (85.7*u* − 84) mV can be used to recover the membrane potential in mV.

				
*u*_o_ = 0	$k_{w}^{+}$ =8	$\tau_{v}^{+}$ = 1.4506 ms	*τ*_*fi*_ = 0.10 ms	*D*_∥_ = 0.010 cm^2^/ms
*u*_*u*_ = 1.56	$k_{w}^{-}$ = 20	$\tau_{v1}^{-}$ = 55 ms	*τ*_*si*_ = 2.9013 ms	*D*_⊥_ = 0.003 cm^2^/ms
*u*_*s*_ = 0.9087	*k*_s_ = 2.0994	$\tau_{v2}^{-}$ = 40 ms	*τ*_*s*1_ = 2.7342 ms	—
*u*_*so*_ = 0.65	*k*_*so*_ = 2	$\tau_{w1}^{+}$ = 175 ms	*τ*_*s*2_ = 2 ms	—
$u_{w}^{-}$ = 0.00615	*θ*_*v*_ = 0.3	$\tau_{w1}^{-}$ = 40 ms	*τ*_*so*1_ = 40 ms	—
$u_{w}^{+}$ = 0.0005	$\theta_{v}^{-}$ = 0.2	$\tau_{w2}^{+}$ = 230 ms	*τ*_*so*2_ = 1.2 ms	—
$w_{\infty }^{*}$ = 0.78	*θ*_*w*_ = 0.13	$\tau_{w2}^{-}$ = 115 ms	*τ*_*o*1_ = 470 ms	—
	*θ*_o_ = 0.006	$\tau_{w\infty }$ = 0.0273 ms	*τ*_*o*2_ = 6 ms	—

As detailed in Section 3.4, the generalization to heterogeneous modeling by data assimilation is obtained by imposing a spatial variation of selected parameters, opportunely sorted around their reference values (i.e., Table 1) based on experimentally informed profiles. On this basis, the heterogeneous model is obtained by perturbing parameters of the homogeneous model so that global features in the evoked electrical activity are still correctly reproduced. We numerically integrated the model with an explicit Euler scheme implemented in Fortran, discretizing the spatial operators to account for heterogeneous diffusion and phase-field boundary conditions. We solved the model in both 1D and 2D domains, assuming zero-flux boundary conditions. Space and time discretization is Δ*x* = 0.025 cm, Δ*t* = 0.01 ms. Stability and conduction velocity convergence was verified upon mesh refinement and testing higher-order discretization schemes in time (second- and fourth-order Runge–Kutta), achieving non-significant variations in the computed results.

## 3 Results

Alternans in cardiac tissues is observed at high-frequency entrained (Gizzi et al., 2013; Loppini et al., 2021) and self-sustained freely rotating and heterogeneity-bound spiral waves (Hörning et al., 2017). In the past, those dynamics were difficult to visualize without the use of heavy spatial–temporal filters and thus hindering the fine-scale visualization and study of nodal lines that are observed in discordant alternans. Here, we apply the spatial-filter–free FFI analysis method that was recently introduced by Hörning et al. (2017). It is worth mentioning that unique alternative methods assessing cardiac alternans are still required due to the critical differences in electrophysiological signals. The action potential amplitude (Chen et al., 2017) and calcium transient duration and amplitude (Clusin, 2008; Visweswaran et al., 2013) represent, in fact, different approaches that require a meticulous comparison.

### 3.1 Alternans in Intact Canine Ventricles

**FIGURE 1:**
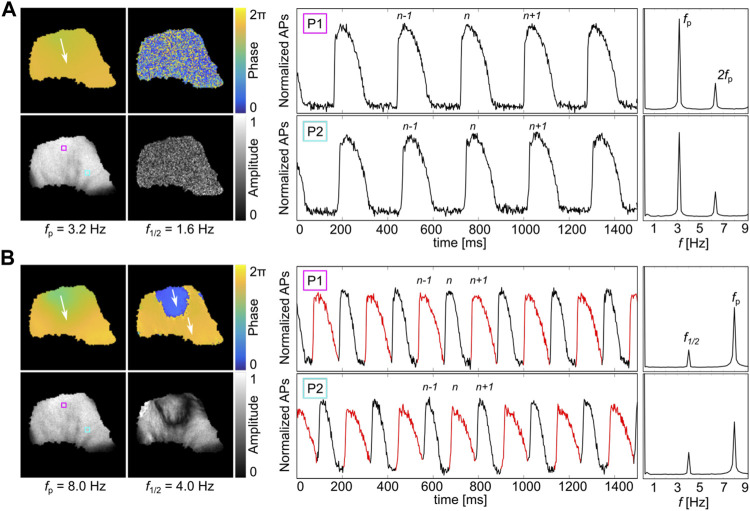
Fourier analysis in a high-frequency entrained canine heart. **(A,B)** show the epicardium of a canine heart that is paced from the top (RV anterior) entrained with *f*_p_ = 3.2 Hz (no alternans) and *f*_p_ = 8.0 Hz (2:2 DA), respectively. Shown is the Fourier space (phase and amplitude) of frequencies *f* and *f*_1/2_. The white arrows indicate the direction of the propagation wave. Two AP rhythms measured at two independent locations (P1 and P2, 6 × 6 pixel FOV) and their respective Fourier spectra are shown exemplarily. **(B)** shows a typical example where nodal lines are visible at *f*_1/2_. Every second AP time course is shown in red to facilitate visualization of the 2:2 AP rhythm. The second peak 2*f*_p_ in the Fourier space of the upper AP rhythms is a typical higher-order frequency mode. The positions P1 and P2 are highlighted by pink and cyan squares in **(A)** and **(B)**. Three waves are marked by the wave numbers, as *n* − 1, *n*, and *n* + 1.

Simultaneous recordings of the epicardium and endocardium in RV canine preparations were observed (Gizzi et al., 2013). Physiological alternans-free wave conduction and alternans states could be observed depending on the pacing site position and pacing frequency. At lower pacing frequencies, no alternans is observed, as the APD is sufficiently short to prevent the interaction of subsequent waves. Figure 1A shows such an example observed at the entrainment frequency *f*_p_ = 3.2 Hz on the epicardium. The phase and amplitude of the pixel-wise FFI-analyzed recordings show a continuously evolving phase and amplitude in the entire tissue. No spatially correlated phase or amplitude is observed at *f*_1/2_ = *f*_p_/2 = 1.6 Hz that would indicate alternans. Also, closer inspection of the local signaling does not indicate alternans (Figure 1A, right panels). The normalized APs at P1 (pink square, top) and P2 (cyan square, bottom) show no alteration in peak height or APD, as confirmed by the respective amplitude in the Fourier space. Only a single peak at the entrainment frequency *f*_p_ is observed. The lower amplitude peak at 2*f*_p_ shows the second mode and does not carry relevant information. Contrarily, alternans is observed when the epicardium is entrained with a higher frequency. Figure 1B shows the same analysis and local signaling recordings at *f*_p_ = 8.0 Hz. In this case, the pacing frequency is sufficiently high so that subsequent waves influence each other. The phase and amplitude information at *f*_1/2_ = 4 Hz shows a typical pattern of discordant 2:2 alternans. That means that two subsequent waves lead to two different APDs in time. In space, the APD of each wave can transiently switch between the two APDs that are spatially confined by nodal lines. The latter can be identified by the spatial phase jump of about *π*, and the amplitude valley. The normalized APs at P1 (cyan) and P2 (pink) show APD alternation between shorter and longer APDs. Every second AP is shown in either black or red to facilitate visualization. For those time series, a second peak at *f*_1/2_ = 4 Hz is visible in the amplitude spectrum, since a second underlying frequency is present that correlates with two times the wave period (2*T* = *f*_1/2_).

### 3.2 Visualization of Higher-Order Discordant Alternans

**FIGURE 2:**
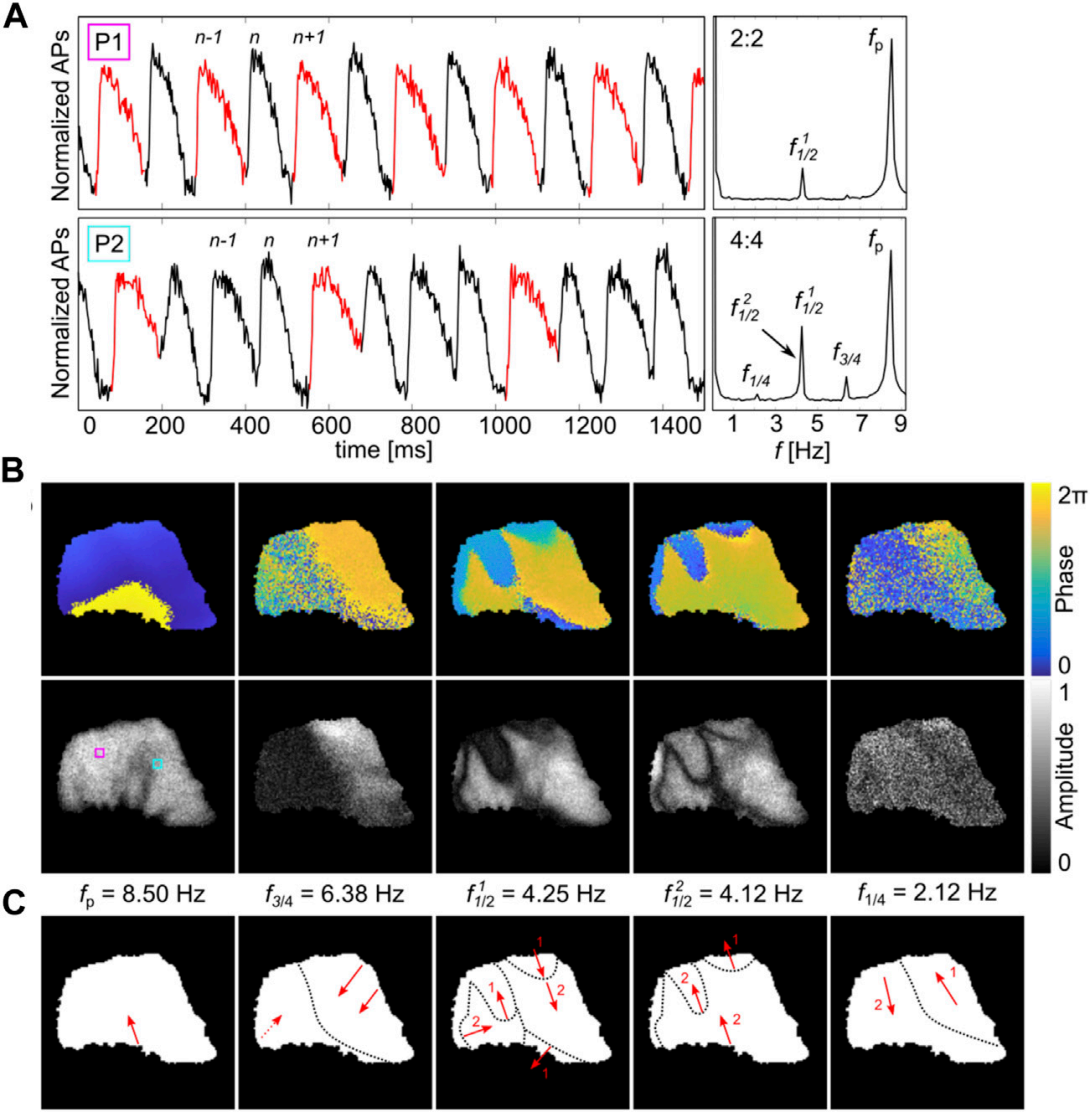
Simultaneous DA of different AP rhythms in a high-frequency entrained epicardium of a canine heart. **(A)** shows two AP rhythms (P1 and P2, 6 × 6 pixel FOV) of 2:2 and 4:4 alternans and their Fourier spectra, respectively. The stimulation site is on the bottom (base) of the heart with *f*_p_ = 8.50 Hz. *f* indicates the entrainment frequency, and *f*_3/4_ indicates the presence of a 4:4 AP rhythm with its two corresponding peaks, $f_{1/2}^{1}$ and $f_{1/2}^{2}$ around *f*_p_/2. The peak at *f*_1/4_ corresponds to a wavelet of four APs that is fully expressed by *f*_p_. Every second or fourth AP is shown in red to facilitate visualization of the 2:2 or 4:4 AP rhythms. **(B)** shows the corresponding spatial phase and amplitude information of the five frequency peaks. The Fourier data shown at *f*_3/4_ indicate a 4:4 AP rhythm on the right side (RV anterior) of the heart only. The respective counter phase is illustrated at $f_{1/2}^{1}$ . $f_{1/2}^{2}$ shows the typical 2:2 phase of a 2:2 AP rhythm, similarly to that illustrated in Figure 1B. The positions P1 and P2 are highlighted by pink and cyan squares. **(C)** shows the direction of the respective phase information indicated by red arrows that are shown in **(B)**. Nodal lines are outlined by black dotted lines. Numbers 1 and 2 shown in $f_{1/2}^{1}$ and $f_{1/2}^{2}$ indicate the phase waves that are shifted by *π*.

Although 2:2 alternans is the most commonly observed AP rhythm, other higher-order AP rhythms exist (see, e.g., Figure 6 in Gizzi et al. (2013)). The epicardium that is shown in Figure 1 was additionally paced from the bottom (base) of the heart at *f*_p_ = 8.5 Hz, which led to a spatially mixed mode of 2:2 and 4:4 alternans (Figure 2A). While the 2:2 AP rhythm shows a single amplitude peak at *f*_1/2_ = 4.25 Hz, two additional amplitude peaks are observed in the Fourier space for the 4:4 AP rhythm: one very close to *f*_1/2_ and one at *f*_3/4_. As the two peaks at around *f*_1/2_ are very close to each other but implicate different information, they are from here on defined as $f_{1\text{/}2}^{1}$ and $f_{1\text{/}2}^{2}$ . The well-defined spatial distribution of the two different AP rhythms is visualized at the phase and amplitude reconstructions in Figure 2B. Here, $f_{1\text{/}2}^{2}$ and *f*_3/4_ show additional spatial information to the frequencies at *f*_p_ and $f_{1\text{/}2}^{1}$ in the Fourier space. The 2:2 (left side, RV anterior) and 4:4 (right side, RV anterior) alternans regimes are spatially separated at *f*_3/4_. The right side shows synchronized phase and correspondingly elevated amplitude signaling that is present on the left side. At $f_{1\text{/}2}^{1}$ and $f_{1\text{/}2}^{2}$ , more complex phase dynamics are observed in addition to the nodal lines that separate different entrained alternating regions in the tissue. Figure 2C shows the corresponding phase wave directions. Numbers 1 and 2 indicate the temporal order of occurring phase waves. In this framework, nodal lines and nodal areas carry more information compared to the classically analyzed temporal differences in APD (Pastore et al., 1999). At the phase of $f_{1\text{/}2}^{1}$ (top, right side, Figure 2B), the phase wave accelerates at the nodal line from 1 to 2 (Figure 2C), while contrarily at $f_{1\text{/}2}^{2}$ , the phase waves propagate in the opposite direction and jump by 2*π* (top, right side, Figure 2B). The latter indicate the typical phase waves, as observed in the Fourier space $\left(f_{1\text{/}2}^{2}\right)$ at 2:2 AP rhythms (see also Figure 1). For the sake of completeness, Figure 2 shows additionally *f*_1/4_ that corresponds to the duration of four AP rhythms. However, *f*_1/4_ is a rather non-significant frequency and appears only because of the intrinsic noise of the system, as the peak at *f*_p_ describes already the main oscillation of the system, and the three other amplitude peaks at $f_{1\text{/}2}^{1}$ , $f_{1\text{/}2}^{2}$ , and *f*_3/4_ describe the periodic temporal variations.

### 3.3 Spatial Synchronization of Alternans Patterns

**FIGURE 3:**
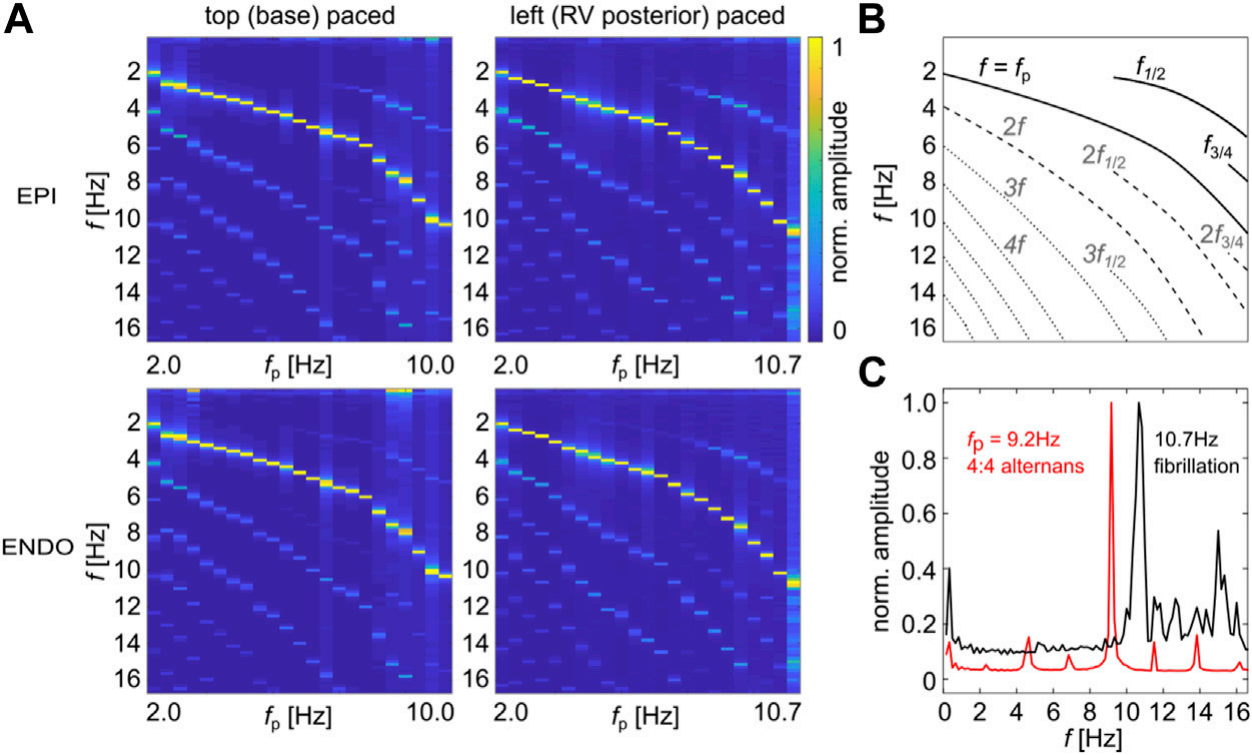
Pacing-site–dependent frequency maps. **(A)** shows frequency maps obtained from the top (base, left panels) and left (RV posterior, right panels) paced canine ventricles, respectively. The top and bottom panels show data recorded at the epicardium (EPI) and endocardium (ENDO). 2:2 AP rhythms (*f*_1/2_) are observed from about 4.5 Hz. 4:4 AP rhythms (*f*_3/4_) are observed only in base paced canine recordings at a pacing of about 9.2 Hz. **(B)** shows a guide of the eye for **(A)** with the main frequencies (solid lines), secondary peaks (dashed lines), and higher-ordered peaks (dotted lines). **(C)** shows a comparison of the normalized amplitudes for 4:4 alternans (9.2 Hz, red line) and fibrillation (10.7 Hz, black line) that is observed at the ENDO paced from the RV posterior.

The frequency response observed at a single recorded pixel is useful to get an overview of the local alternans offset (in analogy to the well-known restitution curves). Figure 3A shows frequency maps with the normalized amplitudes depending on the entrainment *f*_p_ for top (base, left panels) and left (RV posterior, right panels) paced canine ventricles. The top and bottom panels show data recorded at the epicardium (EPI) and endocardium (ENDO). The main peaks (bright yellow peaks) indicate *f*_p_. Above *f*_p_ appears a second peak from about 5 Hz that indicates alternans $\left(f_{1\text{/}2}^{2}\right)$ . Additionally, only for very high frequencies, here *f*_p_ = 9.2 Hz, a peak at *f*_3/4_ is visible that indicates 4:4 alternans. Figure 3B shows a generalized scheme of the frequency maps for comparisons. The offset of 2:2 alternans differs between the EPI and the ENDO, while the peak *f*_3/4_ is observed for all four frequency maps at the same entrainment frequency *f*_p_ = 9.2 Hz. At frequencies higher than 10.0 Hz, fibrillation is observed, as additionally shown in the right (*f*_p_ = 10.7 Hz, RV posterior) paced EPI and ENDO frequency maps. The comparison between *f*_p_ = 9.2 Hz and 10.7 Hz is shown in Figure 3C. Fibrillation shows an elevated baseline and a broad spectrum for frequencies above *f*_p_.

**FIGURE 4:**
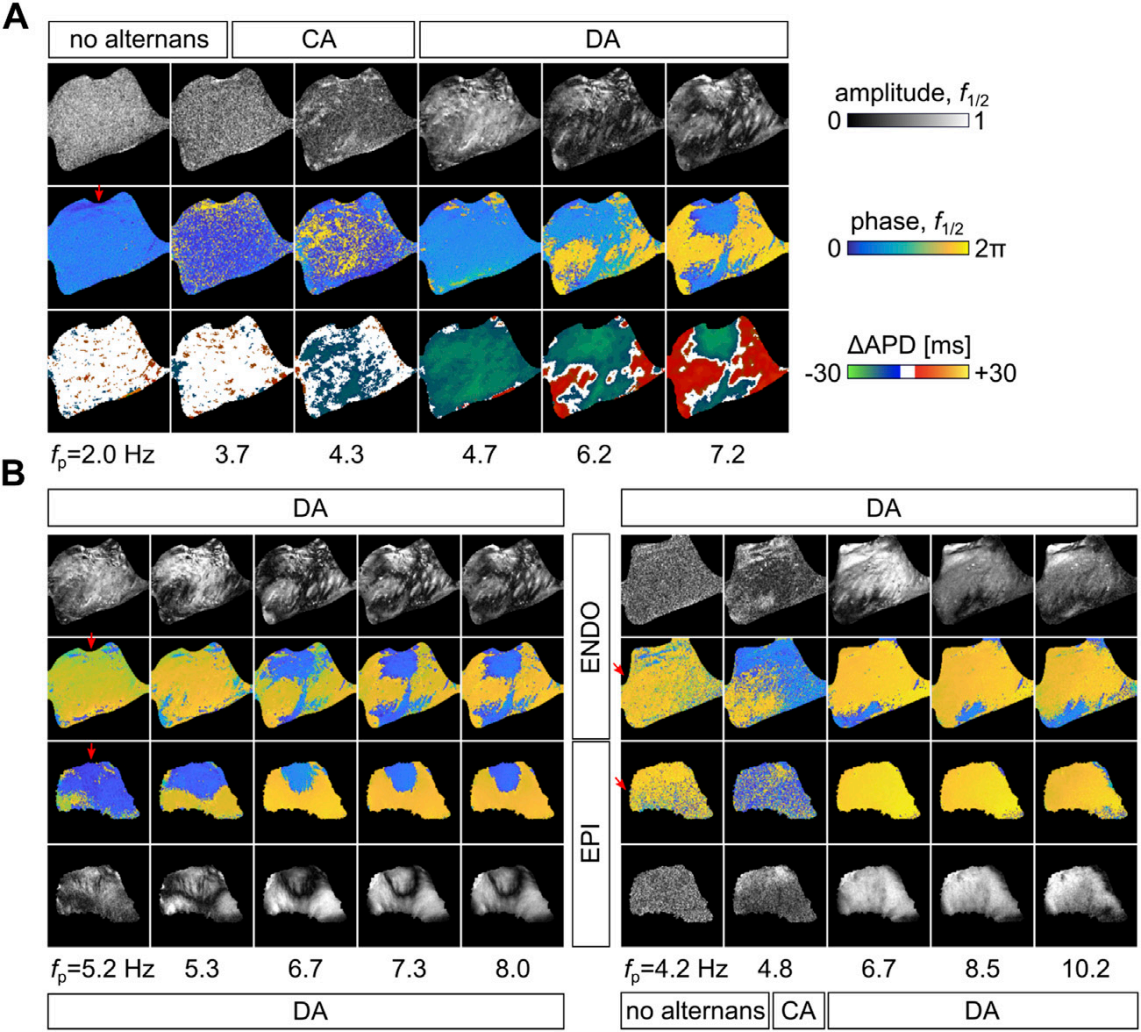
Stabilization of nodal line formation at higher entrainment frequencies. **(A)** shows the evolution of alternans from lower to higher pacing frequencies. The Fourier space, phase and amplitude at *f*_1/2_ and ΔAPD, is shown from *f*_p_ = 2 Hz to 7.2 Hz. **(B)** shows the concordant alternans evolution of the frequency maps shown in Figures 3A,B. The top (base, left panels) and left (RV posterior, right panels) paced canine hearts are shown on the left and right sides, and the respective epicardium (EPI) and endocardium (ENDO) are shown at the bottom and top. The regimes of no alternans, concordant alternans (CA), and discordant alternans (DA) are indicated on the top and bottom of the figures. Red arrows indicate the position of the electrode.

While a critical frequency induces fibrillation, the complex spatiotemporal alternans patterns stabilize with the increasing entrainment frequency (Gizzi et al., 2013) (Figure 4A). Figure 4B shows selected snapshots of the EPI and ENDO from two different pacing sites. Initially, no alternans is observed at the EPI at a lower *f*_p_ ≃ 4 Hz, but the initiation of alternans at the ENDO can be seen (endocardium base paced, Figure 4). Interestingly, this occurs in larger speckled patches rather than in defined areas, which indicates the alternans-offset difference among individual cells. This speckled-like early fine-scale initiation of alternans was suggested previously by Jia et al. (2010). With increasing *f*_p_, those patterns synchronize spatially and lead to distinct phase areas that are spatially separated by nodal lines, as best visible at the EPI. Although the ENDO shows comparable stabilization of alternans in the phase, the amplitude shows more spatial variations. This is most likely caused by the influence of the Purkinje fibers that are confined to the subendocardial layer and believed to be responsible for the initiation of ventricular fibrillation (Fox et al., 2002; Muñoz et al., 2018).

### 3.4 Data Assimilation From Optical Ultrastructure

**FIGURE 5:**
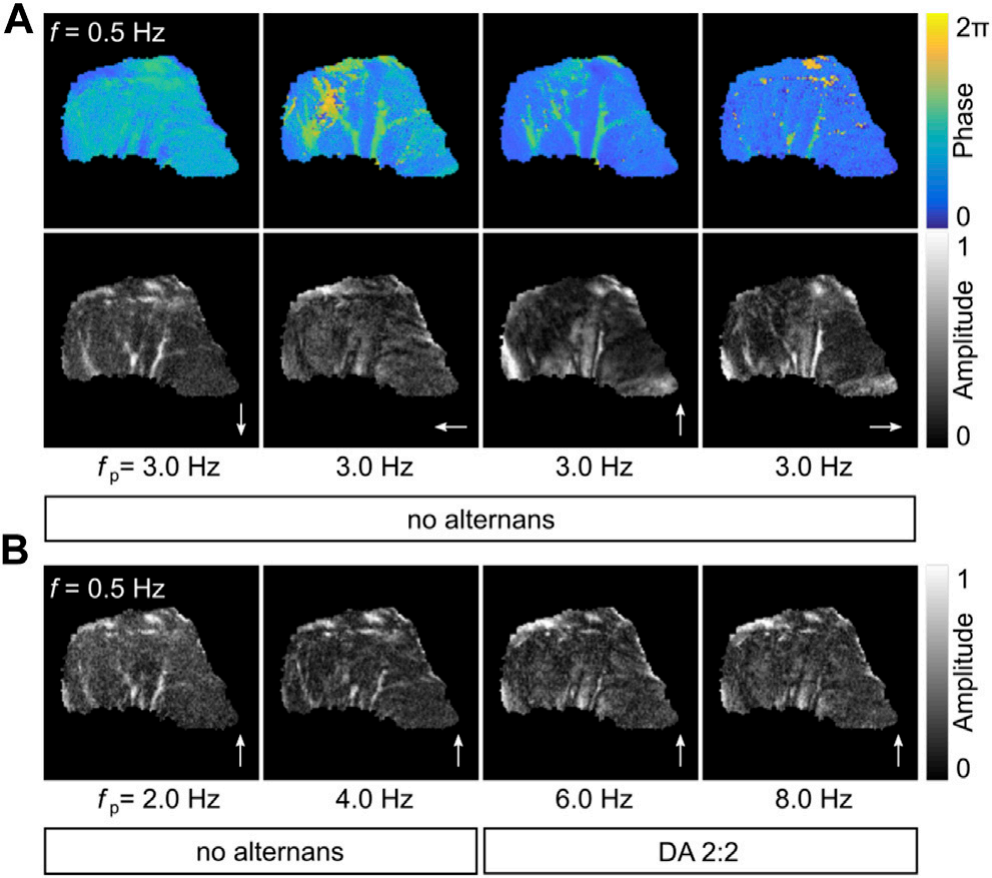
Optical ultrastructure extracted from the low-frequency regime of the epicardium (EPI). **(A)** shows the Fourier space—phase and amplitude—at *f* = 0.5 Hz for *f*_p_ = 3 *Hz* frequency entrained tissues that are paced on the endocardium from four different directions, as indicated by the white arrows. **(B)** shows the amplitudes of the Fourier space at *f* = 0.5 Hz for different entrainment frequencies *f*_p_ that are stimulated at the base. The white arrows in **(A,B)** indicate the respective direction of wave propagation. Below the amplitude images are indicated the regimes of no alternans and discordant 2:2 alternans (DA).

As the differences of the electrophysiological properties of individual cells also lead to differences in the alternans-offset and restitution characteristics, it is useful to take pixel-based differences into account when modeling alternans dynamics *in silico*. The advantage of the Fourier analysis of heart tissues is that the optical ultrastructure can be revealed in the low-frequency regime, as shown in Figure 5. Especially, the amplitude information at *f* = 0.5 Hz is a stable indicator for morphologically restricted differences that are independent of the pacing location (Figure 5A) and pacing frequency (Figure 5B). Here, we utilize the low-frequency regime, as it is also an indirect measure of the signal height, i.e., the observed baseline of the AP rhythms. Therefore, we assume that the strength of the emitted signal depends on the local tissue properties and thus relates to the heart ultrastructure.

In order to validate this hypothesis, we propose a novel data assimilation approach using the ultrastructure observed at *f* = 0.5 Hz assuming the influence in the diffusive term, Eq. 2.3 (*D*_∥_, *D*_⊥_), and the time constants that shape the AP, Eq. 5 ( $\tau_{w}^{+}$ , *τ*_*so*_, and *τ*_*si*_). The tissue heterogeneities are applied on a pixel-based scale, so that the local variations of conduction velocity and action potential are accounted through specific parameters having a strong impact on the resulting APD. In detail, a mask encoding the actual tissue of the experimental samples was extracted by analyzing the signal-to-noise ratio, and the spatial map of the Fourier amplitude spectrum at *f* = 0.5 Hz was computed. The resulting ultrastructure profile was smoothed by using a Gaussian kernel, restricted to a radius of 6 pixels with a variance equal to 5. The heterogeneity field was evaluated by normalizing the ultrastructure mask as

$$H\left(x,y\right)=\delta \;\frac{r}{max\left(\left|r\right|\right)}+1,$$ (8)

where $r=s-\bar{s}$ , *s* = |*F*_*x*,*y*_ (0.5 Hz)|, and *δ* is the parameter denoting the desired maximal variation. We set this value in the range [0,1] by assuming heterogeneity variations of the local parameters up to 100%. Eventually, the heterogeneity map is combined with the tissue map to include also information on tissue boundaries (i.e., phase-field). We refer to this heterogeneity field as an *H*_1_(*x*, *y*) map. Furthermore, we considered a second heterogeneity field that enhances the data assimilation procedure from low-amplitude areas in the Fourier spatial map. We evaluated the reciprocal of the *H*_1_ (*x*, *y*) map and normalized the result according to Eq. 8, still constraining the parameter *δ* ∈ [0, 1]. This second spatial scalar field is denoted as the *H*_2_(*x*, *y*) map with average 1 and variation *δ*. It is worth noting that the proposed procedure represents a generalization of the phase-field method (Fenton et al., 2005) that permits both the inclusion of tissue boundary and specific modulations of model parameters. The resulting heterogeneity maps are used as a multiplication factor on selected model parameters to achieve a constitutive heterogeneity shaped on a tissue ultrastructure. In our analyses, we focused on heterogeneities related to diffusivity and APD parameters (*D*_∥_, *D*_⊥_, $\tau_{w}^{+}$ , *τ*_*so*_, *τ*_*si*_), by using the following constitutive law:

$$p\left(x,y\right)=\bar{p}\;H_{i}\left(x,y\right).$$ (9)

Here, *p* (*x*, *y*) denotes a spatial dependent parameter, $\bar{p}$ is the original model parameter, and *H*_*i*_(*x*, *y*) is the heterogeneity field (with *i* = 1, 2). A visual representation of the adopted data assimilation technique is thoroughly provided in the next sections. Naming *P*_1_ as the set of diffusivity parameters and *P*_2_ as that of APD-regulating time constants, we investigated all possible combinations of *H*_1_ and *H*_2_ maps on *P*_1_ and *P*_2_, i.e., a specific heterogeneity profile was applied to diffusivities and APD-related time constants. It is worth noting that, with this setting, we are assuming a correlation between diffusivity and APD, with such parameters non-linearly coupled through complex electronic effects in cardiac tissue.

### 3.5 *In Silico* Data Assimilation and Alternans Model Prediction

We performed an extensive *in silico* study on both one-dimensional cables and two-dimensional tissues to test the heterogeneity effects on alternans onset and severity. In particular, we computed *H*_1_ (*x*, *y*) and *H*_2_ (*x*, *y*) maps from a selected experimental tissue to shape model parameters’ heterogeneity in 1D and 2D domains, investigating all possible combinations of the heterogeneity fields on diffusivity and APD-regulating time constants, at *δ* = 0.25, 0.5, 1. For 1D simulations, we extracted one-dimensional cuts of *H*_1_ (*x*, *y*) and *H*_2_ (*x*, *y*) maps along the experimental propagating wavefronts (not shown). This preliminary set of numerical simulations was used as a first benchmark of the data assimilation procedure. We observed that the heterogeneous model is able to 1) recover the expected average CV and AP features and 2) emphasize alternans onset and severity, also inducing conduction block phenomena not observed in the homogeneous case. We then tested data assimilation within 2D computational domains observing notable differences with respect to the homogeneous case. In the following, we show two representative examples comparing the overall results for the same ventricle stimulated with a pacing-down protocol both at the ventricle base and in the right anterior ventricular region. The pacing-down protocol consists in stimulating the tissue starting from a low frequency and progressively reducing the pacing frequency. In particular, at each frequency, we delivered a stimulation train of 10 beats to ensure the tissue reached a stationary regime. This protocol reproduces the experimental one, and in our analyses, we computed alternans patterns on the last two beats to avoid transient effects.

#### 3.5.1 Base Ventricle Stimulation

**FIGURE 6:**
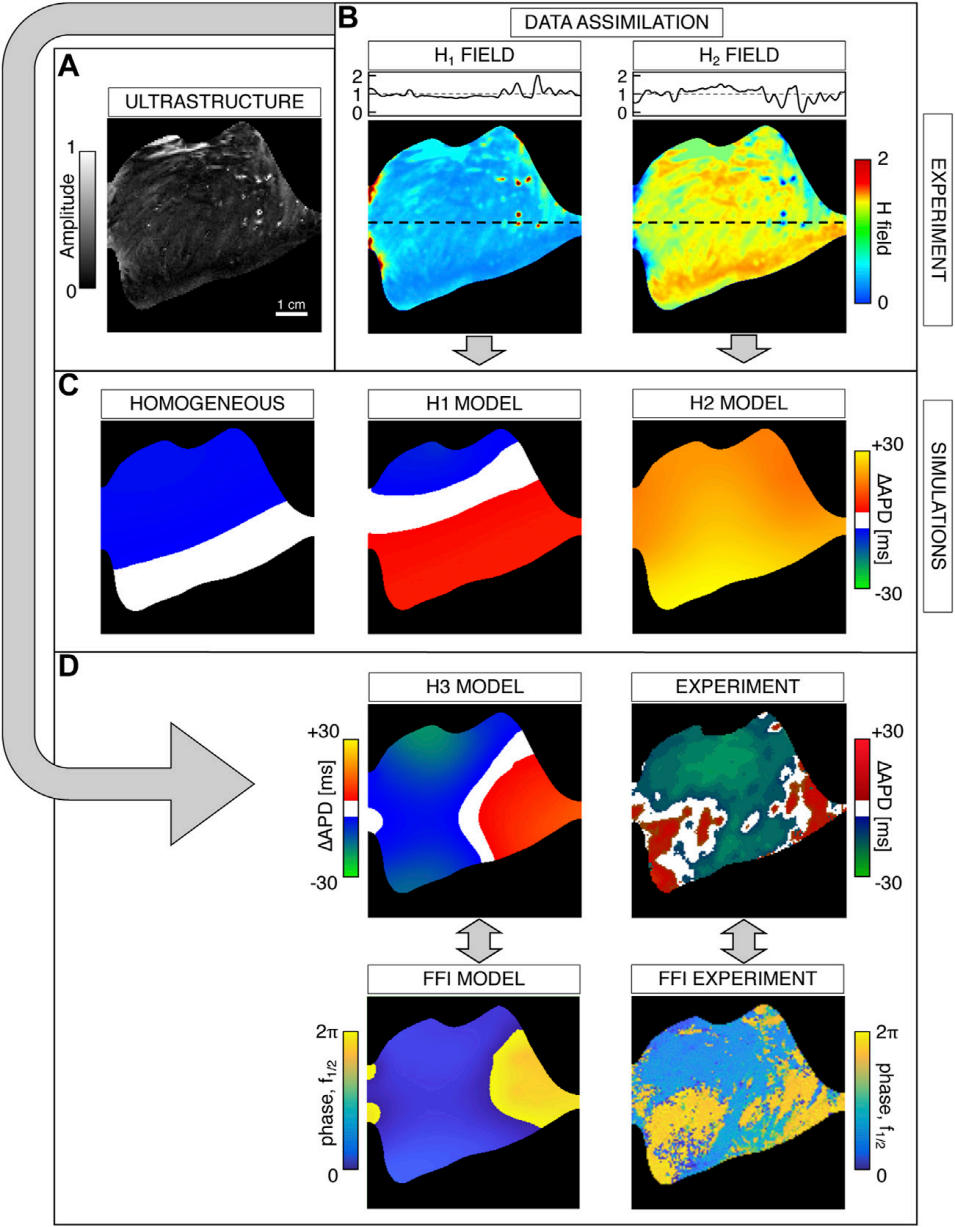
Data assimilation procedure and cardiac alternans maps for stimulation at the base of the ventricle. **(A)** Spatial map of the Fourier spectrum at *f* = 0.5 Hz and tissue boundary. **(B)** Computed heterogeneity maps from the tissue ultrastructure (see the text) for both diffusivity, *H*_1_ (*x*, *y*), and time constants regulating the APD, *H*_2_ (*x*, *y*). Black dashed lines represent one-dimensional cuts of the heterogeneity maps (top panels). **(C)** Modeled alternans maps: homogeneous case, heterogeneity in diffusivity (H1 model), and heterogeneity in APD (H2 model). **(D)** Comparison between the modeled alternans map, with combined heterogeneities in APD-regulating time constants and diffusivity (H3 model), and experimental alternans. Top row: ΔAPD maps. Bottom row: FFI phase maps at *f* = *f*_*p*_/2 (*f*_1/2_). Alternans maps are obtained at a pacing frequency *f*_*p*_ = 6.2 ± 0.4 Hz.

The phase-field ultrastructure and heterogeneity maps are shown in Figures 6A,B. In this case, we tested the model with 1) spatially homogeneous parameters, 2) *H*_1_ maps applied on diffusivity (H1 model), 3) *H*_2_ maps applied on APD-regulating parameters (H2 model), and 4) *H*_1_ and *H*_2_ maps applied simultaneously (H3 model). Figure 6C shows simulated alternans maps for a selected frequency *f*_*p*_ = 6.2 ± 0.4 Hz. On the left, the homogeneous model could not reproduce complex and discordant alternans maps during the pacing-down protocol. Both H1 and H2 models (center and right columns) succeeded in reproducing transition into the discordant alternans regime, though showing regular spatial boundaries. Interestingly, the H2 model produced multiple transitions between concordant and discordant alternans during pacing-down (Supplementary Figure S1). However, such a high number of transitions are not observed in experimental activation maps, suggesting that the H2 model is not the optimal choice. The optimal match was finally obtained with the H3 model, capable of recovering a consistent number of CA-DA transitions and complex alternans patterns, i.e., irregular nodal line shape (Figure 6D). The accuracy of the model was also checked by comparing the FFI phase maps computed at *f* = *f*_*p*_/2 (*f*_1/2_). In particular, the *π*-out-of-phase regions of the simulated tissue recovered the shapes obtained with the standard ΔAPD analysis. As detailed in previous paragraphs, such a phase shift is typical of 2:2 alternans. This result shows the applicability of the FFI phase maps on *in silico* data as well to reveal DA spatial patterns and also confirms the accuracy of the data assimilation model in reproducing experimental activation maps.

#### 3.5.2 Anterior Right Ventricle Stimulation

**FIGURE 7:**
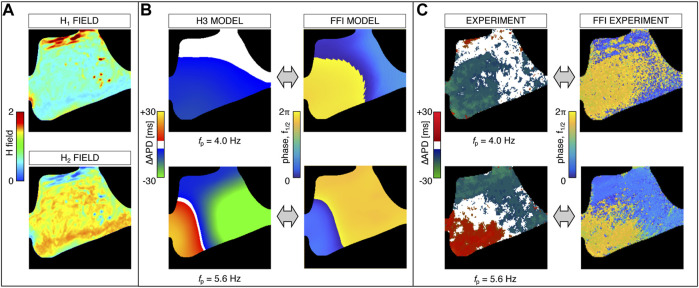
Data assimilation and cardiac alternans maps for right ventricle anterior stimulation. **(A)** Heterogeneity maps for both diffusivity, *H*_1_ (*x*, *y*), and time constants regulating the APD, *H*_2_ (*x*, *y*). **(B)** Modeled alternans maps at pacing frequencies *f*_*p*_ = 4.0 Hz and 5.6 Hz and corresponding FFI phase maps at *f* = *f*_*p*_/2 (*f*_1/2_). **(C)** Experimental alternans corresponding to modeled maps shown in panel **(B)** and corresponding experimental FFI phase maps at *f*_1/2_.

We further investigated the H3 model behavior in response to anterior right ventricle stimulation. The adopted heterogeneity maps for this case are shown in Figure 7A. During pacing-down, the model reproduced both CA and DA alternans patterns as well as multiple transitions between the two regimes. Figure 7B shows simulation results at two pacing frequencies, *f*_*p*_ = 4.0 Hz and *f*_*p*_ = 5.6 Hz, corresponding to two representative cases of CA and DA maps characterized by complex alternans patterns. Also in this case, FFI phase maps at *f*_1/2_ extracted from simulated data are in agreement with the ΔAPD maps and further verify the accuracy of the method in grasping both CA and DA patterns. In particular, CA FFI phase maps show a less severe phase shift compared to the DA case (less than *π*). Indeed, in Figure 7, a change in phase around the blue–yellow transition denotes a minimal phase shift, given the 2*π*-periodicity. In contrast, a phase shift of ≃ *π* arises in the case of DA patterns. As shown in Figure 7C, simulated maps are in close agreement with the experimental ones in terms of both ΔAPD and FFI phase, and similar spatial alternans shapes are recovered both for CA and for DA.

## 4 Conclusion

We have shown that single-pixel Fourier imaging of high-frequency entrained intact canine RV preparations is a valuable tool to visualize action potential alternans. Besides 2:2 DA, as observed in stable spiral waves *in vitro* (Hörning et al., 2017), we have also shown that higher-order DA, e.g., 4:4, can be observed and analyzed in an *ex vivo* heart preparation (Gizzi et al., 2013, 2017). This indicates the possibility of fast and reliable full heart analysis *in vivo* to detect electrical instabilities in cardiac tissues and thus enables the application to the medical field. The unnecessity of spatial filtering of the recorded signals further opens the possibility of detecting ultra-fine structured early alternans that is only restricted by the optical recording device. Contrarily, Fourier imaging needs a specific time window of periodic oscillations to fully take advantage of the Fourier analysis. Subsequent action potentials cannot be compared and visualized as for the established analysis of action potential duration difference, i.e., ΔAPD (see Figure 4B). So, depending on the purpose, Fourier imaging is a powerful alternative to detect and visualize alternans.

A second useful application is the use of the optical ultrastructure that can be extracted in the low-frequency regime in the Fourier space (see Figure 3). As the ultrastructure is related to the morphological properties of the tissue, we assimilated this frequency and pacing site–independent structure to recover alternans *in silico*. Using a phenomenological model tuned on CV and restitution curves (Fenton et al., 2013), we were able to reproduce strikingly similar CA and DA patterns as we have observed experimentally. In this context, experimental tissue heterogeneities included in the model could induce CA–DA transitions and complex shapes of alternating tissue areas and nodal boundary lines, not recovered in the fine-tuned homogeneous model. Furthermore, our analysis proved the FFI method to be a practical approach to uncover alternans on *in silico* data, showing phase maps in close agreement with ΔAPD dispersion.

Pros and cons of the present study shall be mentioned. As for the data assimilation, alternative methods can be used for parameter inference. Genetic algorithms or variational approaches aim at fitting recorded spatiotemporal cardiac activity targeting diffusive properties encoded in the conductivity tensor (Cairns et al., 2017; Barone et al., 2020b; Irakoze and Jacquemet, 2021). If these methods mostly work in the time domain, the data assimilation technique here proposed focuses on the frequency domain instead. It allows, in fact, to account for changes in cardiac tissue properties not considered in other parameter fitting techniques. We believe that our method, combined with other procedures, can enrich data assimilation toward customized models with high predictive power. The present numerical model, in fact, was limited to two-dimensional computational domains (though based on ventricular geometries). An additional level of predictability is expected to appear once the whole ventricular thickness is considered. In such a scenario, the mathematical characterization of intramural rotational anisotropy, combined with a surface-based FFI data assimilation, may open the path toward a multiscale assessment and control of alternans, as well as to scale-transitioning information theories (Garzón et al., 2009; Ashikaga and James, 2018).

We remark that various approaches could be used to derive heterogeneity fields from the FFI spectrum. Indeed, slight variations in the selected frequency or alternative transformation laws can lead to different *H* (*x*, *y*) maps. In the present study, we performed a specific choice considering the invariance of the emergent FFI spatial structure and the non-random organization of the amplitude dispersion. Besides, the adopted scaling can be interpreted as a “perturbed” version of the homogeneous model allowing investigating heterogeneity effects without additional parameter optimization. Although different interpretations of FFI peaks and valleys can be pursued to derive optimized heterogeneity maps and maximize data assimilation, we remark that the present method is generally applicable to multiple cardiac surfaces (endocardium–epicardium, atria–ventricles) and integrated with both biophysical and phenomenological models. Furthermore, one can sort parameters other than diffusivity and APD-regulating time constants based on heterogeneity fields and pursue different assumptions on their correlation. In this context, we assumed that diffusivity and APD-regulating time constants followed correlated spatial heterogeneity profiles. Accordingly, we developed our investigation on this hypothesis as a first explorative study on the effect of a frequency-based data assimilation procedure on cardiac modeling. We hope our study could be further tested and validated in future numerical analyses.

Tissues undergoing fluorescence optical mapping are inherently wet, and they must be kept without drying out to retain physiologically realistic activity. The wet tissue reflects directional light into the camera, causing bright patches in the image known as specular reflection. Regions with specular reflection do not contain information on the tissue texture. Furthermore, these bright spots could produce unrealistic distortion due to the change in angle between the surface and the light source during small residual deformations. On the contrary, diffusion only contains the wavelengths that were not absorbed by the tissue and therefore carry texture information. In such a perspective, including specialized lighting setups would concur to reduce specular reflection. In particular, a cross-polarized lighting setup may provide the best quality images with the least specular reflection and most detailed textures (Lentle and Hulls, 2018). The appearance of optical ultrastructure further connects the present study with a major and multidisciplinary research effort in high-resolution imaging of large biological tissues (Kuruppu et al., 2021).

To conclude, the FFI method outlined in this contribution represents a new and effective method to investigate alternans onset and development in whole-ventricle optical experiments. Accordingly, it can be potentially applied to both calcium and voltage data and does not require excessive pre-analysis, such as the APD-based approaches. Moreover, we have shown that spectral analysis of experimental data at low frequencies can be used to uncover invariant and coherent spatial structures associated with the underlying cardiac tissue properties—ultrastructure—which can serve as input for data assimilation in numerical simulations.

## Data Availability

The raw data supporting the conclusions of this article will be made available by the authors, without undue reservation.

## Supplementary Material

Supplementary Figure S1 Modeled alternans maps during the pacing-down protocol with stimulation at the base of the ventricle: A) homogeneous model, B) H1 model, C) H2 model, and D) H3 model (see the text). The homogeneous model is not able to reproduce the transition into the DA regime and complex alternans boundaries. As for the H1 and H2 models, the heterogeneity fields are able to induce CA–DA transitions, still not recovering complex shapes of nodal lines. When the heterogeneity fields *H*_1_ and *H*_2_ are appropriately combined in the H3 model to shape, respectively, diffusivity and APD-regulating parameters, both the recovered number of CA–DA transitions and alternating tissue shapes are consistent with the experimental observations.
